# miR-151-3p Targets TWIST1 to Repress Migration of Human Breast Cancer Cells

**DOI:** 10.1371/journal.pone.0168171

**Published:** 2016-12-08

**Authors:** Ting-Chih Yeh, Tzu-Ting Huang, Tien-Shun Yeh, Yu-Ren Chen, Kai-Wen Hsu, Pen-Hui Yin, Hsin-Chen Lee, Ling-Ming Tseng

**Affiliations:** 1 Institute of Anatomy and Cell Biology, School of Medicine, National Yang-Ming University, Taipei, Taiwan; 2 Department and Institute of Pharmacology, School of Medicine, National Yang-Ming University, Taipei, Taiwan; 3 Department of Medical Research, Taipei Veterans General Hospital, Taipei, Taiwan; 4 Department of Surgery, Taipei Veterans General Hospital, and National Yang-Ming University, Taipei, Taiwan; 5 Taipei-Veterans General Hospital Comprehensive Breast Health Center, Taipei, Taiwan; University of Hong Kong, HONG KONG

## Abstract

TWIST1 is a highly conserved basic helix-loop-helix transcription factor that contributes to cancer metastasis by promoting an epithelial-mesenchymal transition and repressing E-cadherin gene expression in breast cancer. In this study, we explored the potential role of miR-151 in TWIST1 expression and cancer properties in human breast cancer cells. We found that the human TWIST1 3’UTR contains a potential binging site for miR-151-3p at the putative target sequence 5’-CAGUCUAG-3’. Using a TWIST1-3’UTR luciferase reporter assay, we demonstrated that the target sequence within the TWIST1 3’UTR is required for miR-151-3p regulation of TWIST1 expression. Moreover, we found that ectopic expression of miR-151-3p by infection with adenoviruses expressing miR-151 significantly decreased TWIST1 expression, migration and invasion, but did not affect cell growth and tumorsphere formation of human breast cancer cells. In addition, overexpression of the protein coding region without the 3’UTR of TWIST1 reversed the repression of cell migration by miR-151-3p. Furthermore, knockdown of miR-151-3p increased TWIST1 expression, reduced E-cadherin expression, and enhanced cell migration. In conclusion, these results suggest that miR-151-3p directly regulates TWIST1 expression by targeting the TWIST1 3’UTR and thus repressing the migration and invasion of human breast cancer cells by enhancing E-cadherin expression. Our findings add to accumulating evidence that microRNAs are involved in breast cancer progression by modulating TWIST1 expression.

## Introduction

Breast cancer is one of the most common malignancies in women and its incidence rate is increasing [1]. Although there has been a remarkable improvement in mortality from breast cancer, recurrence and metastases remain the major causes of death for breast cancer patients [2]. Understanding the mechanisms responsible for breast cancer progression and developing more specifically targeted, less toxic therapies are critical issues in breast cancer treatment.

In human breast cancers, TWIST1 is usually found to be over-expressed, which is correlated with invasive lobular carcinoma, a highly infiltrating tumor type associated with loss of E-cadherin expression, lymph-node and distant metastases, and poor patient prognosis [3–6]. TWIST1 is a highly conserved basic helix-loop-helix (bHLH) transcription factor and is characterized by a basic DNA binding domain that targets the consensus E-box sequence 5’-CANNTG-3’ and a helix-loop-helix domain [7]. TWIST1 contributes to cancer metastasis by promoting an epithelial-mesenchymal transition (EMT) [8, 9]. Moreover, TWIST1 is a transcriptional repressor of E-cadherin gene expression in breast cancer [10]. Based on the function of E-cadherin as a cell-cell adhesion molecule, loss of E-cadherin is considered a pre-requisite for EMT favoring tumor cell dissemination and metastasis [8]. Therefore, the regulation of TWIST1 expression in cancer cells might be a potential target for the suppression of cancer cell metastases.

MicroRNAs (miRNAs) are endogenous small single-stranded non-coding RNAs, typically 20–22 nucleotides in length, that regulate gene expression by binding specific sequences in the 3’-untranslated region (3’-UTR) of the target mRNA [11, 12]. Accumulating evidence has proven that deregulation of miRNA is involved in a wide range of human diseases, including cancer [13]. In human cancer, miRNAs can function as oncogenes or tumor suppressor genes during tumorigenesis, depending on their target genes [14]. Recently, some miRNAs were identified to modulate cancer properties by directly targeting TWIST1 expression in different cancer cells [15], suggesting that TWIST1 might be regulated by different miRNAs during cancer progression.

In this study, we adopted in silico analyses and found that the TWIST1 3’UTR contains a potential binging site for miRNA (miR)-151-3p at the putative target sequence from nucleotide position (np) 71 to np 87. The miR-151 gene localizes to chromosome 8q24.3 and resides within intron 22 of the host gene encoding focal adhesion kinase (FAK) [16]. It has been reported that miR-151 regulates tumor cell migration and spreading of hepatocellular carcinoma (HCC) [16, 17]. Downregulating Rho GDP Dissociation Inhibitor (GDI) Alpha (RhoGDIA) by miR-151 enhanced HCC cell migration through the activation of Rac1, Cdc42 and Rho GTPases [16]. In breast cancer, miR-151-5p expression levels were not different among tumors of varying grades, but the level was significantly lower in the lymph-node metastases than in their corresponding tumors of breast cancer patients [18]. It was recently demonstrated that miR-151-5p combined with other miRNAs (miR-145a-5p or miR-337-3p) are able to significantly repress TWIST1 translation and result in the decreased migratory potential of murine embryonic fibroblast cells [19]. However, the role of miR-151 in breast cancer progression and its direct targets in the regulation of breast cancer metastasis are still undefined. In this study, we explored the potential role of miR-151 in TWIST1 expression and cancer properties in human breast cancer cells.

## Materials and Methods

### Plasmids and plasmid construction

The DNA sequence of the human TWIST1 3’-UTR (nucleotide positions 961–1247 from the start of the 5’-UTR) was amplified by polymerase chain reaction (PCR) from HEK293T cells using the primers TW-3UTR-f: 5’-ATC TCG AGC AGG CGG AGC CCC CCA-3’ and TW-3UTR-r: 5’-ATG CGG CCG CCA ACT GTT CAG ACT TCT ATC-3’. The PCR products were digested with *Xho*I and *Not*I, and they were then ligated into the reporter vector psiCHECKTM-2 (TWIST1 3’-UTR-WT). To create an 8-bp deletion (miR-151-3p target sequence, 5’-CAGTCTAG-3’) within the TWIST1 3’UTR, the primer pair TW-3UTR-f and 3UTR-Rd-r (5’-CAG AGT CTT CCA TTT TCT CCT TCT C-3’) and the primer pair 3UTR-Fd-f (5’-AAA ATG GAA GAC TCT GGA GCT GGA TA-3’) and TW-3UTR-r were used to generate two PCR fragments lacking the miR-151-3p target sequence. The two PCR fragments were used to generate the TWIST1 3’-UTR 8bp-deletion by PCR using the primers TW-3UTR-f and TW-3UTR-r. The PCR products were digested with *Xho*I and *Not*I and were then ligated into the reporter vector psiCHECKTM-2 (TWIST1 3’-UTR-8bp-deletion). The overexpression construct pFLAG-TWIST1 (pTWIST1) containing the TWIST1 protein coding region without the 3’UTR and pFLAG-CMV (empty vector, pEV) was a gift from Prof. Muh-Hwa Yang at National Yang-Ming University [20].

### Cell culture

The cell lines MCF7, MDA-MB-231, HCC1937, and HEK293T were obtained from the American Type Culture Collection (ATCC). The MCF7, MDA-MB-231, and HEK293T cells were grown in Dulbecco’s modified Eagle’s medium (Gibco, Carlsbad, USA) supplemented with 10% fetal bovine serum (FBS). Human breast cancer HCC1937 cells were cultured in RPMI 1640 (Gibco, Carlsbad, USA) supplemented with 10% fetal bovine serum. The cells were incubated in a humidified atmosphere of 5% CO_2_ and 95% air at 37°C.

### Transfection

The cells were transiently transfected with TurboFect or Lipofectamine^™^ 2000 (Invitrogen) transfection reagent. For the reporter assay, HEK293T or MDA-MB-231 cells (4 × 10^5^) were seeded into 6-well plates. After infection with adenoviruses expressing miR-151 (Ad-miR-151) or GFP (Ad-GFP), the cells were transiently transfected with psiCHECKTM-TWIST1-3’UTR-WT, psiCHECKTM-TWIST1- 3’UTR-8bp-deletion, or an empty plasmid using the TurboFect reagent. For overexpression of TWIST1, pFLAG-TWIST (pTWIST1) or the empty vector (pEV) were transfected into MDA-MB-231 cells by TurboFect after infection with adenoviruses expressing miR-151 (ad-miR-151) or GFP (ad-GFP). For knockdown of miR-151-3p, antagomir-151-3p or scrambled negative control oligonucleotides (Ambion) were transfected into HCC1937 cells at a final concentration of 100 nM using Lipofectamine^™^ 2000 (Invitrogen), respectively.

### Recombinant adenoviruses

The recombinant miR-151-expressing adenoviral plasmid was linearized by *Pac*I and subsequently transfected into the adenovirus packaging cells AD-293 to obtain packaged recombinant adenovirus expressing miR-151 (designated Ad-miR-151). The pAdTrack-CMV empty vector was used to generate recombinant adenovirus expressing GFP as a control (designated Ad-GFP). The titer and multiplicity of infection of recombinant adenoviruses were determined according to the manufacturer’s protocol (Stratagene).

### Luciferase reporter assay

HEK293T or MDA-MB-231 cells (4 × 10^5^) were infected with adenoviruses expressing miR-151 or GFP for 24 hours. After transfection using TurboFect with psiCHECKTM-TWIST1-3’-UTR-WT or psiCHECKTM-TWIST1-3’-UTR-8bp- deletion plasmids for 24 hours, the cell extracts were prepared, and the luciferase activity was measured using the Dual-Luciferase Reporter Assay System (Promega).

### Quantitative real-time PCR analysis

Total RNA was isolated using Trizol reagent (Invitrogen, Carlsbad, CA), and cDNA was synthesized using Moloney murine leukemia virus reverse transcriptase (New England BioLabs, Beverly, MA) with an oligo (dT_18_) primer. Quantitative real-time PCR was performed using a Light Cycler system with a LightCycler FastStart DNA Master PLUS SYBR Green I (Roche, Indianapolis, IN) [21]. The primers used in real-time PCR analysis are TWIST1-f: 5'-GGG AGT CCG CAG TCT TAC GA-3', TWIST1-r: 5'-AGA CCG AGA AGG CGT AGC TG-3'; β-catenin-f: 5'-CCA GCC GAC ACC AAG AAG-3', β-catenin-r: 5'-CGA ATC AAT CCA ACA GTA GCC-3'; E-cadherin-f: 5'-CCC ACC ACG TAC AAG GGT C-3', E-cadherin-r: 5'-CTG GGG TAT TGG GGG CAT C-3'; N-cadherin-f: 5'-CCA TCA AGC CTG TGG GAA TC-3', N-cadherin-r: 5'-GCA GAT CGG ACC GGA TAC TG-3'; Slug-f: 5'-TGT GTG GAC TAC CGC TGC-3', Slug-r: 5'-TCC GGA AAG AGG AGA GAG G-3'; Snail-f: 5'-ACA TCC GAA GCC ACA CGC TGC-3', Snail-r: 5'-CGC AGG TTG GAG CGG TCA GC-3'; Vimentin-f: 5'-CCT TGA ACG CAA AGT GGA ATC-3', Vimentin-r: 5'-GAC ATG CTG TTC CTG AAT CTG AG-3'; and GAPDH-f: 5'-CCG TCT AGA AAA ACC TGC C-3', GAPDH-r: 5'-GCC AAA TTC GTT GTC ATA CC-3'. The relative mRNA levels were normalized to that of GAPDH. For the detection of mature miRNAs, cDNA synthesis and TaqMan miRNA real-time PCR assays were performed as previously described [22, 23]. The relative miRNA levels were normalized to the level of RNU48, which is a small nucleolar RNA.

### Western blot

Whole cell extracts were prepared using radioimmunoprecipitation assay (RIPA) buffer (150 mM NaCl, 50 mM Tris-HCl, 0.1% SDS, 0.5% sodium deoxycholate, 0.1% Triton X-100) plus 10 μg/ml aprotinin, 2 mM EGTA, 2 mM Na_3_VO_4_ and 1 mM PMSF. The protein concentrations were determined using the Bradford assay (Sigma-Aldrich), and the samples were diluted in 5X Laemmli buffer (300 mM Tris-HCl pH 6.8, 10% SDS, 5%, 2-mercaptoethanol, 25% glycerol, 0.1% bromphenol blue) and boiled for 5 min. Proteins (40 μg) were separated by 8~15% SDS-PAGE and transferred onto polyvinylidene fluoride (PVDF) membranes (Pall Life Sciences). Non-specific binding sites on the PVDF membranes were blocked with 5% non-fat milk in TBST (20 mM Tris-HCl, pH 7.6, 137 mM NaCl, 1% Tween-20). The membranes were then hybridized with primary antibodies overnight at 4°C, followed by incubation with horseradish peroxidase (HRP)-conjugated secondary antibodies. The membranes were then developed using Immobilon Western Chemiluminescence HRP Substrates (Millipore). The images were captured using a Luminescence/Fluorescence Imaging System (GE Healthcare), and the signal intensities were quantified using Multi Gauge image analysis software (Fujifilm).

### Cell proliferation assay

Cell proliferation was determined using the MTT assay. The cells (3 × 10^4^) were seeded in 24-well plates cultured overnight prior to infection. After infection with adenoviruses expressing miR-151 or GFP for 48 hours, the medium was discarded and replaced with 0.25 mg/ml 3-[4,5-dimethylthiazol-2-yl]-2,5-diphenyl-tetrazolium bromide (MTT; Sigma-Aldrich) and incubated for 2 hours at 37°C in the dark. The reagent was discarded, and the plates were washed with PBS. The cells were combined with 250 μl dimethyl sulfoxide (DMSO) to dissolve the formazan produced. The absorbance at 550 nm was measured using a microplate enzyme-linked immunosorbent assay reader (TECAN Infinite 200). We further determined cell growth using the sulforhodamine B (SRB) assay. After infection with adenoviruses expressing miR-151 or GFP for 48 hours, MDA-MB-231 cells (5 × 10^3^) were seeded in 96-well plates for 0, 24, 48, 72 and 96 hours. The cells were fixed with 10% ice-cold trichloroacetic acid (TCA) (Sigma-Aldrich) at 4°C for 1 hour, rinsed four times with distilled water and air dried. The cells were then stained with 0.057% SRB (Sigma-Aldrich) in 1% acetic acid for 30 min at room temperature. After rinsing four times with 1% acetic acid and air drying, 50 μl of 10 mM Tris-base (pH 10.5) was added to each well for 30 min. The colorimetric level was read using a microplate reader (Tecan) at 510 nm.

### Tumorsphere formation assays

HCC1937 cells (2 × 10^3^ cells) were suspended in serum-free Dulbecco’s modified Eagle’s medium containing 1% N2, 2% B27, 20 ng/ml human fibroblast growth factor-2 and 20 ng/ml epidermal growth factor (Invitrogen) and subsequently seeded into 96-well ultra-low attachment plates (Corning). The cells were incubated at 37°C for 9 days and supplemented with medium every 3 days [24]. Spheres >50 μm in diameter were counted under the microscope.

### Migration and invasion assays

Cellular migration and invasion abilities (1 × 10^4^ for migration assay, 5 × 10^4^ for invasion assay) were analyzed in 24-well plates using Millicell tissue culture plate well inserts (Millipore, Bedord, MA) for 12 hours and BD BioCoat Matrigel Invasion Chambers (Becton Dickson, Mountain View, CA) for 20 hours, respectively. A total of 5 × 10^4^ breast cancer cells were placed in the serum-free medium inside the inserts and the 10% FBS medium was added in the lower chamber. Then, the cells on the upper surface of membrane were removed with a cotton swab after incubation. After fixation with methanol, cells in the lower surface of membrane were stained with 0.005% crystal violet in PBS for 1 hour, and the number of migrated or invaded cells was determined by counting 10 random fields under a microscope.

### Statistical analysis

All data are presented as the mean ± SEM (standard error of the mean) from at least 3 independent experiments. Significant differences between the control and treatment groups were determined using Student’s *t*-test, and the significance was considered at *P* < 0.05.

## Results

### The TWIST1 3’UTR contains a putative miR-151 binding site that is required for miR-151-3p regulation of TWIST1 expression

**Fig 1:**
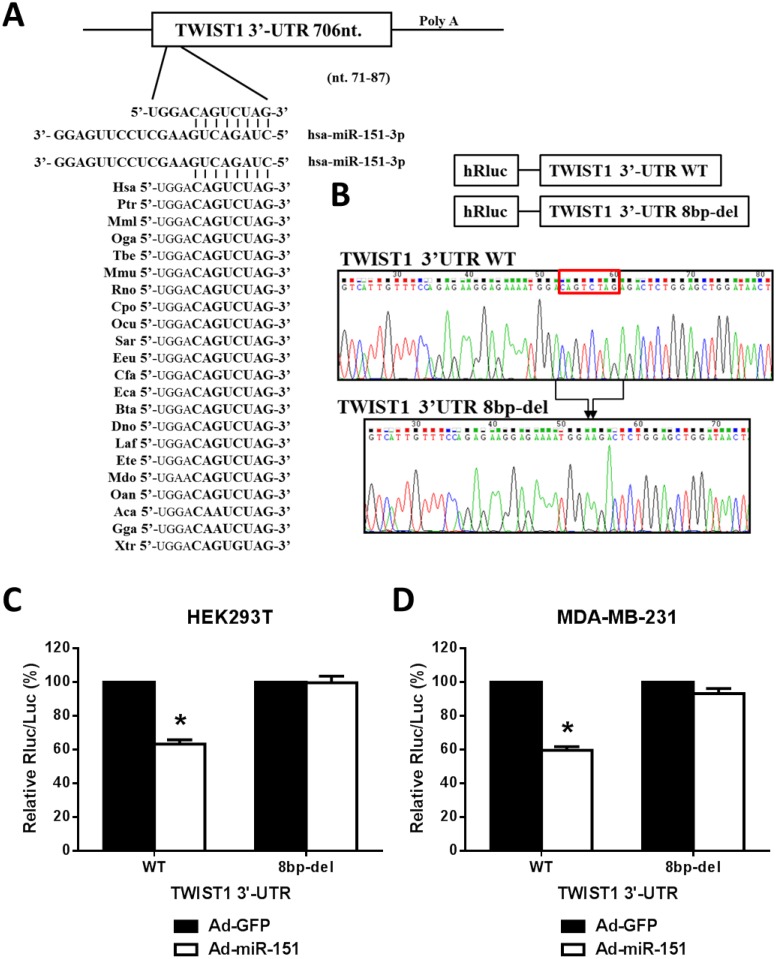
The TWIST1 3’UTR contains a putative miR-151-3p binding site that is required for miR-151-3p regulation of TWIST1 expression. (A) There is a putative miR-151 binding site in the human TWIST1 3’UTR (nucleotide position 71 to 87) as predicted by DIANAmT, miRanda, miRDB, miRWalk, PICTAR5 and Targetscan. The sequences containing the miR-151 binding site of the TWIST1 3’-UTRs of different species are aligned, including Has/ Human/ Homo sapiens (Symbol/ Common name/ Species); Ptr/ Chimpanzee/ Pan troglodytes; Mml/ Rhesus/ Macaca mulatta; Oga/ Bushbaby/ Otolemur garnetti; Tbe/ Treeshrew/ Tupaia belangeri; Mmu/ Mouse/ Mus musculus; Rno/ Rat/ Rattus norvegicus; Cpo/ Guinea pig/ Cavia porcellus; Ocu/ Rabbit/ Oryctolagus cuniculus; Sar/ Shrew/ Sorex araneus; Eeu/ Hedgehog/ Erinaceus europaeus; Cfa/ Dog/ Canis familiaris; Fca/ Cat/ Felis catus; Eca/ Horse/ Equus caballus; Bta/ Cow/ Bos taurus; Dno/ Armadillo/ Dasypus novemcinctus; Laf/ Elephant/ Loxodonta africana; Ete/ Tenrec/ Echinops telfairi; Mdo/ Opossum/ Monodelphis domestica; Oan/ Platypus/ Ornithorhynchus anatinus; Aca/ Lizard/ Anolis carolinensis; Gga/ Chicken/ Gallus gallus; and Xtr/ Frog/ Xenopus tropicalis. (B) The wild type (WT) TWIST1 3’UTR reporter plasmid (TWIST1-3’UTR-WT) and the deleted mutant TWIST1 3’UTR reporter plasmid (TWIST1-3’UTR-8bp-deletion) that lacked the miR-151-3p target sequence 5’-CAGTCTAG-3’were constructed and confirmed by DNA sequencing. (C) After infection with adenoviruses expressing miR-151 (Ad-miR-151) or GFP (Ad-GFP) for 24 hours, HEK293T or MDA-MB-231 cells (4 × 10^5^) were transfected with psiCHECKTM-TWIST1-3’-UTR-WT or psiCHECKTM-TWIST1-3’-UTR-8bp-deletion for luciferase reporter assay. All experiments were performed in triplicate. Each bar represents the mean ± SEM (standard error of the mean). *, *P* < 0.05.

By in silico analyses using predictive software, including DIANAmT, miRanda, miRDB, miRWalk, PICTAR5 and Targetscan, we found that the human TWIST1 3’UTR contains a potential binging site for miR-151-3p at the putative target sequence from nucleotide position (np) 71 to np 87, 5’-CAGUCUAG-3’ (Fig 1A). Importantly, there is a phylogenic conservation of the putative miR-151-3p binding site, the 8 bases 5’-CAGUCUAG-3’, within the TWIST1 3’-UTRs among different mammalian species (Fig 1A), which suggests that TWIST1 expression could be regulated by miR-151-3p.

To validate whether the identified binding site is important for miR-151-3p regulation of TWIST1 expression, we constructed two luciferase reporter plasmids, the wild type TWIST1 3’UTR (TWIST1-3’UTR-WT) and the 8 bp-deletion mutant (TWIST1-3’UTR-8bp-deletion). The two reporter plasmids were successfully constructed and confirmed by DNA sequencing (Fig 1B). Using the two luciferase reporter constructs, we observed that the ectopic expression of miR-151 (Ad-miR-151) can significantly decrease the reporter signals of the TWIST1-3’UTR-WT, but not significantly decrease that of the TWIST1-3’UTR-8bp-deletion when compared with the control (Ad-GFP) groups in HEK293T (Fig 1C) and MDA-MB-231 cells (Fig 1D). These results indicate that the sequence 5’-CAGUCUAG-3’ within the TWIST1 3’UTR is required for miR-151-3p regulation of TWIST1 expression in human cells.

### miR-151 expression is increased in human breast cancers

**Fig 2:**
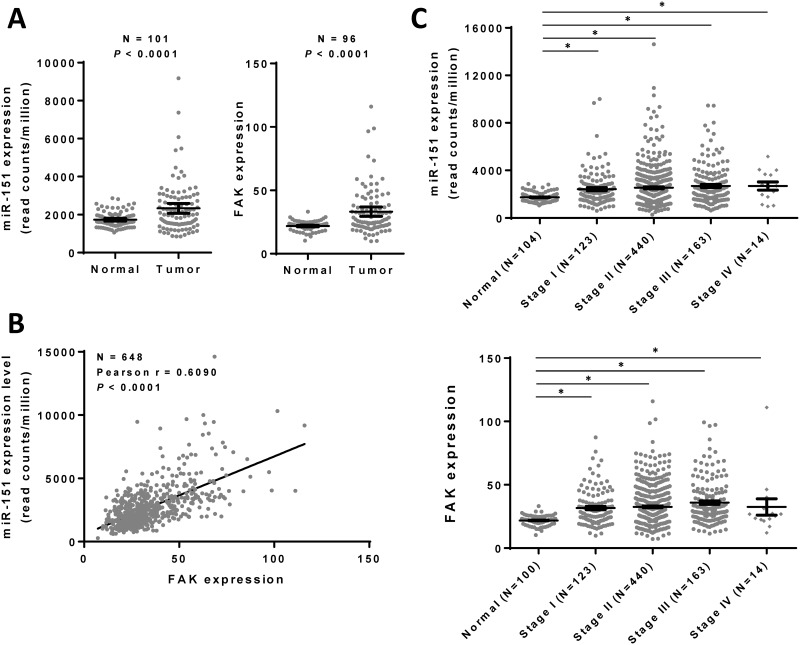
miR-151 expression is higher in breast cancers than in their corresponding adjacent normal tissues. (A) The miR-151 and FAK expression levels of breast cancer samples and the corresponding adjacent normal tissue samples were downloaded from the “The Cancer Genome Atlas” (TCGA) and the Broad GDAC Firehose data portal. The miR-151 reads per million reads of all samples were selected and analyzed to compare abundances. (B) As described above, the miR-151 and FAK expression levels of breast cancer samples (N = 648) were downloaded. The correlation between miR-151 and FAK expression levels was analyzed. (C) As described above, the miR-151 and FAK expression levels of breast cancer samples were downloaded and then divided according to the stage classification. *, *P* < 0.05 as compared with normal breast tissues. The results shown here are reported in their entirety based upon data generated by the TCGA Research Network: http://cancergenome.nih.gov.

To evaluate the miR-151 expression in breast cancers, we analyzed the data obtained from The Cancer Genome Atlas (TCGA) and found that miR-151 expression levels and its host gene FAK mRNA levels in breast cancers were significantly higher when compared with that of paired normal tissues (Fig 2A). Moreover, the miR-151 expression levels were significantly positively correlated with the FAK mRNA levels in breast cancers (Fig 2B). Although no significant difference was observed among the stages of breast cancer, the miR-151 and FAK expression levels of breast cancers at different stages were higher than those of normal tissues (Fig 2C). These results suggest that the miR-151 expression is increased in breast cancer and is associated with the expression of its host gene FAK.

**Fig 3:**
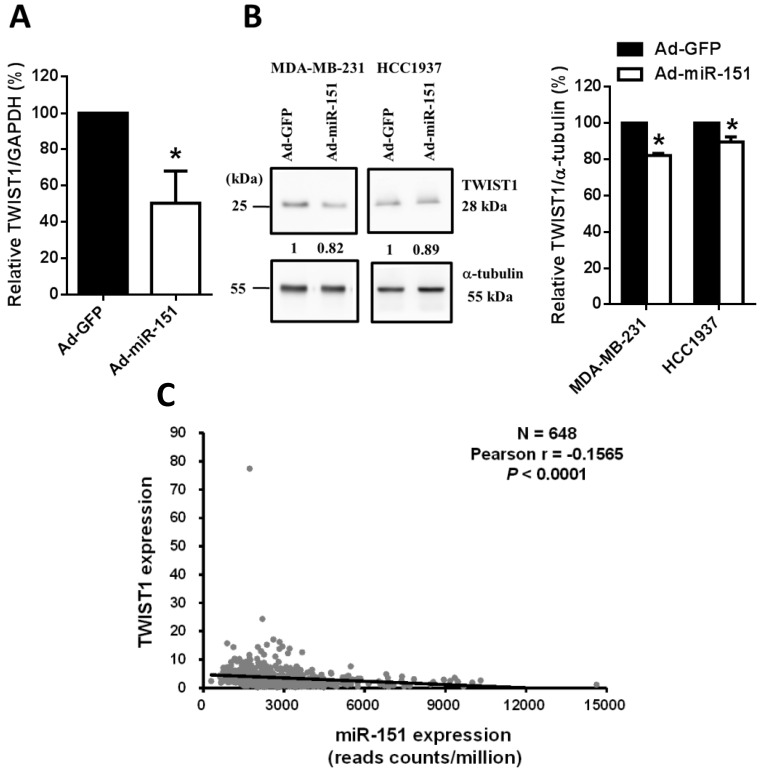
miR-151-3p reduces TWIST1 mRNA and protein expression in human breast cancer cells. (A) After infection with adenoviruses expressing miR-151 (Ad-miR-151) or GFP (Ad-GFP) for 48 hours, the TWIST1 mRNA expression levels of MDA-MB-231 cells were determined using quantitative real-time PCR. The data were normalized to GAPDH mRNA levels. (B) After infection with adenoviruses expressing miR-151 (Ad-miR-151) or GFP (Ad-GFP) for 48 hours, the TWIST1 protein expression levels of MDA-MB-231 cells were determined using Western blotting. The data were normalized to α-tubulin protein levels. All experiments were performed in triplicate. Each bar represents the mean ± SEM (standard error of the mean). *, *P* < 0.05. (C) miR-151 and TWIST1 expression levels of breast cancer samples (N = 648) were downloaded from the “The Cancer Genome Atlas” (TCGA) and the Broad GDAC Firehose data portal. The correlation between miR-151 and FAK expression levels was analyzed. The results shown here are reported in their entirety based upon data generated by the TCGA Research Network: http://cancergenome.nih.gov.

In addition, after infection with adenoviruses expressing miR-151, we found that the mRNA (Fig 3A) and protein (Fig 3B) expression levels of TWIST1 were significantly decreased in human breast cancer cells. The results further confirmed the role of miR-151-3p in regulating TWIST1 expression in human breast cancer cells. Interestingly, it was noted that the data obtained from TCGA showed that there is a significantly negative correlation between the miR-151 expression levels and TWIST1 mRNA levels (Fig 3C). We further evaluated that the correlations between miR-151 and TWIST1-related gene expression levels in human breast cancers and found that miR-151 levels is significantly positive correlated with E-cadherin gene expression levels, and is significantly negative correlated with the gene expressions of fibronectin, osteonectin, and α5-integrin (Table 1). Among these TWIST1-related genes, the expression E-cadherin is repressed by TWIST1, while the expressions of fibronectin, osteonectin, and α5-integrin are activated by TWIST1 [25]. These results suggest that miR-151-3p might regulate TWIST1 expression and thereby TWIST1-related downstream gene expression.

**Table 1:** Correlation of miR-151 and TWIST1-related gene expressions in human breast cancers.

N = 648	miR-151		TWIST1	
TWIST1-related genes	Pearson *r*	*P* value	Pearson *r*	*P* value
TWIST1	-0.1565	< 0.0001*	-	-
Repression by TWIST1				
Claudins	0.0320	0.4158	-0.0368	0.3495
Occludin	0.0600	0.1272	-0.0687	0.0807
E-cadherin	0.1116	0.0044*	-0.0305	0.4380
Desmoplakin	0.0722	0.0660	-0.0514	0.1909
Plakoglobin	0.0716	0.0687	-0.0530	0.1775
Activation by TWIST1				
Fibronectin	-0.2108	< 0.0001*	0.1270	0.0012
Vitronectin	-0.0274	0.4870	0.0288	0.4647
N-cadherin	-0.0241	0.5405	0.0482	0.2205
Osteonectin	-0.3172	< 0.0001*	0.2064	< 0.0001*
α5-integrin	-0.2221	< 0.0001*	0.1812	< 0.0001*

The expression levels of miR-151 and TWIST1-related genes of breast cancer samples (N = 648) were downloaded from the “The Cancer Genome Atlas” (TCGA) and the Broad GDAC Firehose data portal. The correlation between miR-151 and TWIST1-related gene expression levels was analyzed.

*, *P* < 0.05. The results shown here are reported in their entirety based upon data generated by the TCGA Research Network: http://cancergenome.nih.gov.

### miR-151-3p represses cell migration and invasion, but not cell growth and tumorsphere formation of human breast cancer cells

**Fig 4:**
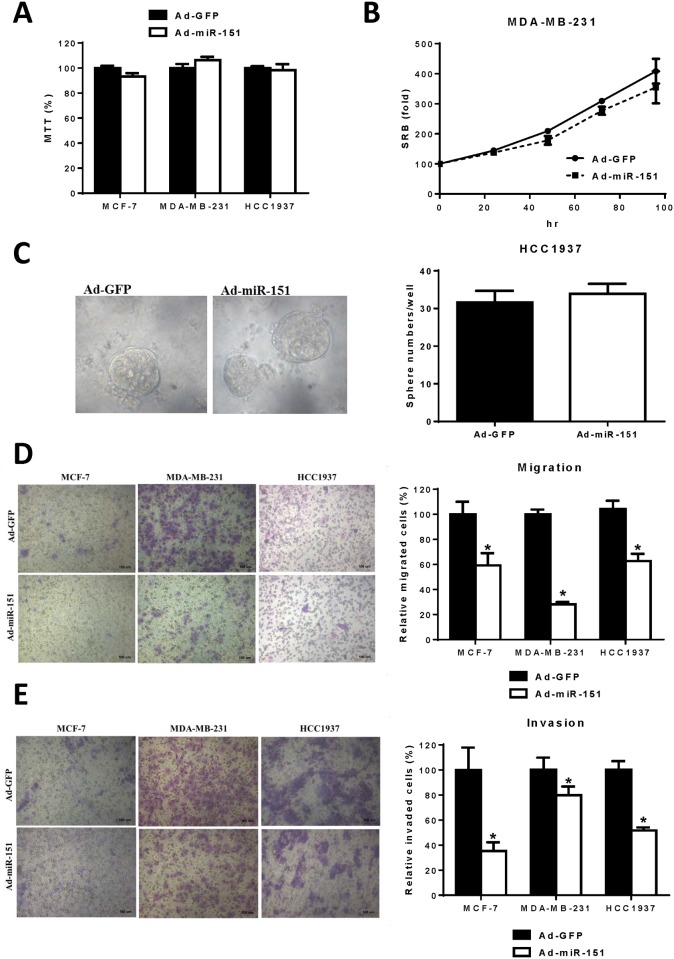
miR-151-3p represses cell migration and invasion, but not cell growth and tumorsphere formation of human breast cancer cells. (A) MCF-7, MDA-MB-231 and HCC1937 cells (3 × 10^4^) were infected with adenoviruses expressing miR-151 or GFP for 48 hours. The viable cells were measured using MTT assay. (B) After infection with adenoviruses expressing miR-151 or GFP for 48 hours, MDA-MB-231 cells (5 × 10^3^) were seeded on 96-well plates for 0, 24, 48, 72 and 96 hours. The relative cell numbers were measured using SRB assay. (C) After infection with adenoviruses expressing miR-151 or GFP for 48 hours, HCC1937 cells (2 × 10^3^) were seeded for a tumorsphere formation assay. After 9 days of incubation, spheres >50 μm in diameter were counted under the microscope. (D) After infection with adenoviruses expressing miR-151 or GFP for 48 hours, MCF-7, MDA-MB-231 and HCC1937 cells (1 × 10^4^) were seeded for transwell migration. After incubation for 12 hours, the migrated cells were counted from 10 random fields under a microscope. (E) After infection with adenoviruses expressing miR-151 or GFP for 48 hours, MCF-7, MDA-MB-231 and HCC1937 cells (5 × 10^4^) were seeded for Matrigel invasion. After incubation for 20 hours, the invaded cells were counted from 10 random fields under microscope. All experiments were performed in triplicate. Each bar represents the mean ± SEM. *, *P* < 0.05.

To evaluate the biological function of miR-151-3p in human breast cancer cells, we ectopically expressed miR-151 in three breast cancer cell lines (MCF-7, MDA-MD-231, and HCC1937) by infection with adenoviruses expressing miR-151 and analyzed the effects on cell growth, tumorsphere formation, cell migration and invasion. The results of the MTT assay (Fig 4A) and SRB assay (Fig 4B) revealed that the ectopic expression of miR-151 does not significantly alter cell growth when compared with that of controls. In addition, the number and size of the tumorspheres within HCC1937 cells were not significantly affected by ectopic expression of miR-151 (Fig 4C). Using a transwell migration assay and a Matrigel invasion assay, we found that both migration (Fig 4D) and invasion (Fig 4E) of the three human breast cancer cell lines were significantly repressed by infection with adenoviruses expressing miR-151. These results indicate that miR-151-3p can regulate cell migration and invasion, but not cell growth and tumorsphere formation in human breast cancer cells.

### Overexpression of the TWIST1 protein-coding region reverses miR-151-3p inhibition of cell migration

**Fig 5:**
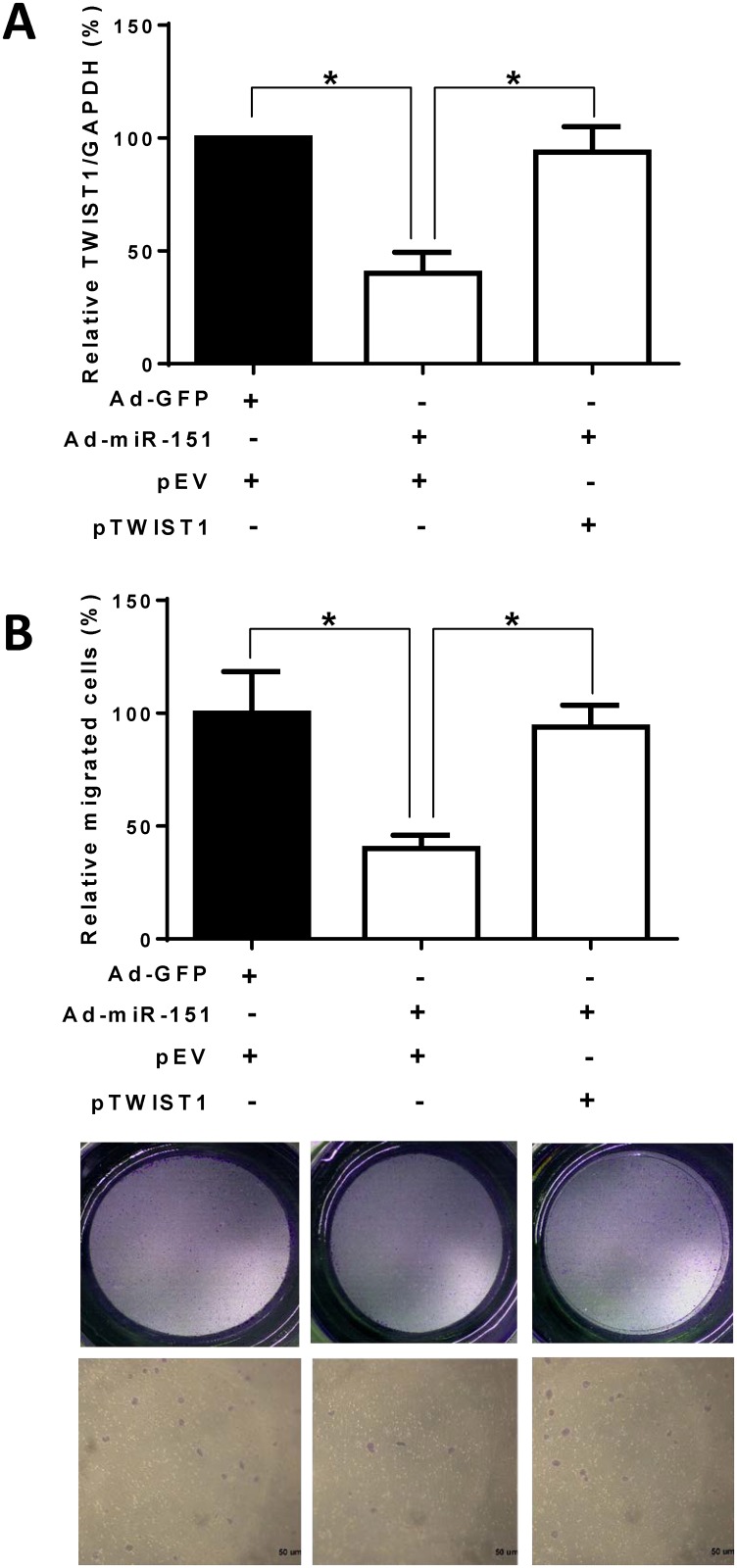
Overexpression of the TWIST1 protein coding region overcomes miR-151-3p inhibition cell migration. (A) After infection with adenoviruses expressing miR-151 (Ad-miR-151) or GFP (Ad-GFP) for 24 hours, MDA-MB-231 cells (4 × 10^5^) were transfected with pEV or pTWIST1. The TWIST1 mRNA expression levels were determined using quantitative real-time PCR. The data were normalized to GAPDH mRNA levels. (B) After infection with adenoviruses expressing miR-151 (Ad-miR-151) or GFP (Ad-GFP) for 24 hours, MDA-MB-231 cells (4 × 10^5^) were transfected with empty vector (pEV) or the overexpression construct pFLAG-TWIST1 that contained the TWIST1 protein coding region (that lacks the TWIST1 3’UTR) (pTWIST1) for 48 hours. The cells (1 × 10^4^) were seeded for transwell migration. After incubation for 12 hours, the migrated cells were counted from 10 random fields under a microscope. All experiments were performed in triplicate. Each bar represents the mean ± SEM. *, *P* < 0.05.

To validate the role of TWIST1 expression in the miR-151-3p regulation of cell migration, we transfected MDA-MB-231 cells with the TWIST1 cDNA plasmid to overexpress the protein coding region (that lacks the TWIST1 3’UTR) after infection with adenoviruses expressing miR-151. We found that overexpression of TWIST1 can reverse the TWIST1 expression that is decreased by ectopic expression of miR-151 (Fig 5A). Importantly, overexpression of TWIST1 can overcome the miR-151’s inhibitory effect on the migration of MDA-MB-231 cells (Fig 5B). These results suggest that miR-151-3p may regulate cell migration through targeting TWIST1 expression.

### Knockdown of miR-151-3p increases TWIST1 expression and enhances cell migration of human breast cancer cells

**Fig 6:**
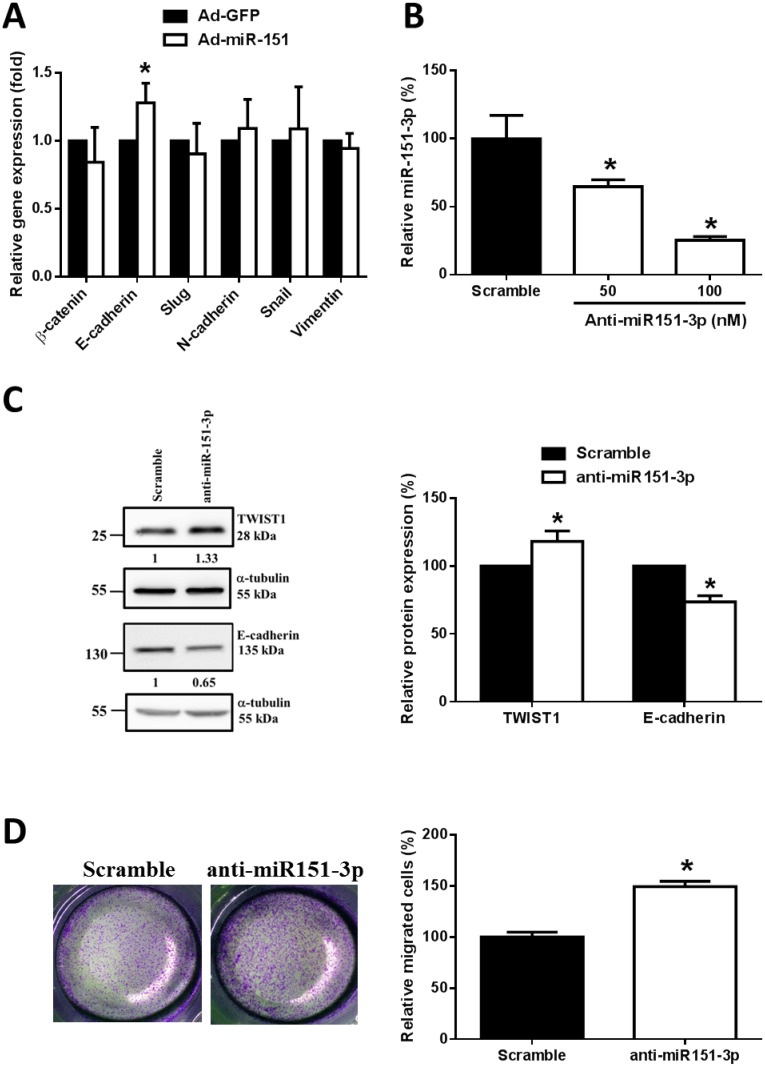
Knockdown of miR-151-3p increases TWIST1 expression and cell migration. (A) After infection with adenoviruses expressing miR-151 (Ad-miR-151) or GFP (Ad-GFP) for 48 hours, the relative gene expression levels of β-catenin, E-cadherin, slug, N-cadherin, snail and vimentin of the MDA-MB-231 cells were measured by quantitative real-time PCR. The data were normalized to GAPDH mRNA levels. (B) HCC1937 cells were transfected with 50 and 100 nM antogomir-151-3p (anti-miR-151-3p) or 50 nM scrambled control for 48 hours. The relative transcript levels of miR-151 were measured by miRNA quantitative real-time PCR. (C) The HCC1937 cells were transfected with 50 nM antogomir-151-3p (anti-miR-151-3p) or 50 nM scrambled control for 48 hours. TWIST1 and E-cadherin protein expression were measured by Western blot analysis. The data were normalized to α-tubulin protein levels. (D) HCC1937 cells were transfected with 50 nM antogomir-151-3p (anti-miR-151-3p) or 50 nM scrambled control for 48 hours. The cells (1 × 10^4^) were seeded for transwell migration. After incubation for 12 hours, the migrated cells were counted from 10 random fields under microscope. All experiments were performed in triplicate. Each bar represents the mean ± SEM. *, *P* < 0.05.

TWIST1 is known as a critical regulator of EMT and is a transcriptional repressor of E-cadherin gene expression in breast cancer. We analyzed the expression of EMT-related genes in MDA-MD-231 cells after infection with adenoviruses expressing miR-151 (Ad-miR-151) and found that the gene expression of E-cadherin was significantly increased, whereas the gene expression levels of β-catenin, slug, N-cadherin, snail and vimentin were not significantly different when compared with that of the control (Ad-GFP) (Fig 6A). The results suggest that E-cadherin may be involved in miR-151-3p-TWIST1-mediated cell migration.

In addition, we used antogomir-151-3p (anti-miR-151-3p) to knockdown endogenous miR-151-3p expression in breast cancer cells. Fig 6B shows that the miR-151-3p expression was significantly decreased by anti-miR-151-3p in a dose-dependent manner. Moreover, knockdown of miR-151-3p increased TWIST1 protein expression and decreased E-cadherin protein expression (Fig 6C). In addition, the migration of HCC1937 cells was significantly increased by anti-miR-151-3p treatment when compared with that of the scrambled control (Fig 6D). These results support the notion that miR-151-3p regulates TWIST1 expression and thus modulates E-cadherin expression as well as cell migration of breast cancer cells.

## Discussion

This is the first study to demonstrate that miR-151-3p directly regulates TWIST1 expression by targeting the TWIST1 3’UTR and thus represses the migration and invasion of human breast cancer cells by enhancing E-cadherin expression. Our findings add to accumulating evidence that microRNAs are involved in breast cancer progression by modulating TWIST1 expression.

**Fig 7:**
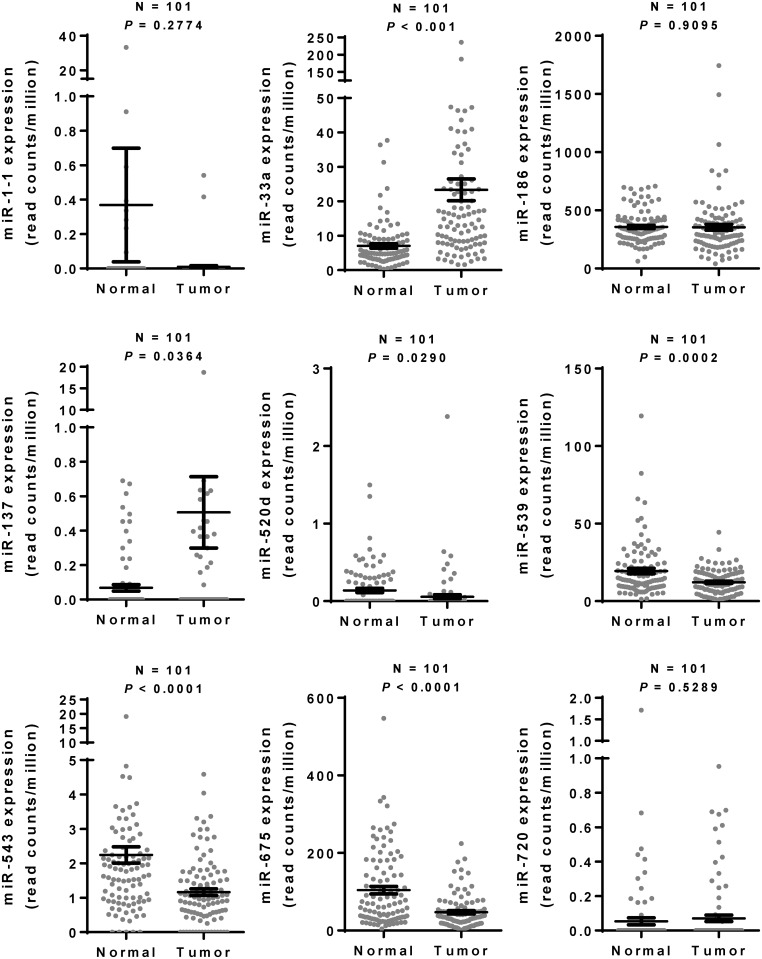
The expression levels of microRNAs in human breast cancers and the corresponding adjacent normal tissues. The expression levels of miR-1-1, miR-33a, miR-137, miR-186, miR-520d, miR-539, miR-543, miR-675, miR-720 in the breast cancer samples and the corresponding adjacent normal tissue samples (N = 101) were downloaded from the “The Cancer Genome Atlas” (TCGA) and the Broad GDAC Firehose data portal. *, *P* < 0.05 as compared with normal breast tissues. The results shown here are reported in their entirety based upon data generated by the TCGA Research Network: http://cancergenome.nih.gov.

Several miRNAs have been documented to directly inhibit TWIST1, including miR-1-1 [26], miR-33a [27, 28], miR-137 [29], miR-186 [30], miR-300 [31], miR-520d-5p [32], miR-539 [31], miR-543 [31], miR-675 [33], and miR-720 [34]. Most of these miRNAs repressed cancer cell EMT and metastasis in most examined cancer types, and some of them (miR-33a and miR-186) modulated cancer cell sensitivity to cisplatin by down-regulating TWIST1 [27, 30]. For example, it was demonstrated that miR-33a is upregulated in chemoresistant osteosarcoma and promotes cell resistance to cisplatin [27]. On the other hand, miR-186 was shown to induce G1 cell-cycle arrest and enhance cell apoptosis, which consequently rendered the cells more sensitive to cisplatin in ovarian cancers [30]. Among these miRNAs, the data obtained from TCGA reveal that like miR-151, miR33a and miR-137 are significantly higher expression levels in breast cancers as compared with the paired normal tissues (Fig 7). The expression levels of miR-520d, miR-539, miR-543, and miR-675 in breast cancers were significantly lower than paired normal tissues (Fig 7). Interestingly, we found that miR-33a and miR-151 are significantly negative correlated with TWIST1 gene expression in breast cancers (Table 2). In the present study, we found that ectopic expression of miR-151 represses cell migration and invasion of breast cancer cells by down-regulating TWIST1 (Figs 4D, 4E and 5B). In addition, knockdown of miR-151-3p by anti-miR-151-3p can up-regulate TWIST1 expression and enhance cell migration (Fig 6D). However, only E-cadherin, but not other EMT-related genes, was significantly up-regulated by increased expression of miR-151-3p, suggesting that E-cadherin might be the major contributor in the TWIST1-promoting cell metastasis that is regulated by miR-151-3p. Moreover, increased expression of miR-151-3p did not affect cell growth and tumorsphere formation of breast cancer cells (Fig 4A, 4B and 4C). These findings suggest that microRNAs might target TWIST1 to regulate different cancer properties in different cancer types, and miR-151-3p might negatively regulate cell metastasis during breast cancer progression.

**Table 2:** Correlations of microRNAs and TWIST1 gene expression in human breast cancers.

miRNA	Pearson *r*	*P* value
miR-1-1	-0.0284	0.4690
miR-33a	-0.1020	0.0093*
miR-137	0.0584	0.1369
miR-186	-0.0736	0.0612
miR-151	-0.1565	< 0.0001*
miR-520d	-0.0192	0.6253
miR-539	0.0068	0.8628
miR-543	0.0652	0.0972
miR-675	-0.0148	0.7078
miR-720	-0.0400	0.3094

The expression levels of TWIST1, miR-1-1, miR-33a, miR-137, miR-186, miR-520d, miR-539, miR-543, miR-675, miR-720 in the breast cancer samples (N = 648) were downloaded from the “The Cancer Genome Atlas” (TCGA) and the Broad GDAC Firehose data portal.

*, *P* < 0.05. The results shown here are reported in their entirety based upon data generated by the TCGA Research Network: http://cancergenome.nih.gov.

The miR-151 gene resides within intron 22 of the FAK gene [16]. The data obtained from TCGA revealed that the miR-151 expression levels in breast cancers are positively correlated with mRNA levels of the miR-151 host gene FAK (Fig 2B). These results suggest that the increased miR-151 expression might be co-regulated with the FAK gene expression in breast cancers. It has been shown that p53 can bind to the FAK promoter and subsequently repress FAK expression. Recently, it was demonstrated that activation of the Notch1 pathway enhances FAK and miR-151 expression [23]. The Notch1 pathway may interact with p53 to regulate FAK and miR-151 expression levels. The mechanism underlying up-regulated expression of miR-151 in breast cancers requires further investigation.

FAK is a key signaling molecule that is activated by numerous stimuli, is often overexpressed in human tumors, and promotes cancer cell invasion and metastasis [35, 36]. FAK and miR-151 have been found to promote cell migration and spreading of HCC [16, 17] and gastric cancers [23]. Although miR-151 expression levels are positively correlated with the FAK mRNA levels in breast cancers (Fig 2B), our results revealed that ectopic expression of miR-151 represses cancer cell migration and invasion (Figs 4D, 4E and 5B), whereas knockdown of miR-151-3p enhances breast cancer cell migration (Fig 6D). These results indicate that unlike in HCC and gastric cancer, miR-151-3p may play a negative regulator in breast cancer metastasis.

Some direct targets of miR-151 have been identified. Downregulation of RhoGDIA by miR-151 was demonstrated to enhance HCC cell migration through activation of Rac1, Cdc42 and Rho GTPases [16]. It was also found that miR-151-3p represses the full-length isoform of human neurotrophin-3 receptor gene NTRK3 in neuroblastoma cells [37]. It was recently demonstrated that miR-151-5p and a combination of other miRNAs (miR-145a-5p or miR-337-3p) are able to significantly repress TWIST1translation and result in decreased migratory potential of murine embryonic fibroblast cells [19]. In the role of miR-151-3p in muscle fiber type determination, miR-151-3p was shown to directly target ATP2a2, a gene that encodes for a slow skeletal and cardiac muscle specific Ca^2+^ ATPase known as SERCA2, thus downregulating slow muscle gene expression [38]. In the present study, we identified TWIST1 as the direct target of miR-151-3p. Our results clearly showed that ectopic expression of miR-151-3p represses TWIST1 expression (Figs 1C, 1D, 3A, 3B and 5A), whereas knockdown of miR-151-3p increases TWIST1 expression (Fig 6C). These results demonstrated that TWIST1 expression is negatively regulated by miR-151-3p. Interestingly, the data obtained from TCGA revealed a significant negative correlation between the TWIST1 mRNA levels and miR-151 expression levels in human breast cancers (Fig 3C). Moreover, miR-151 levels is significantly positive correlated with E-cadherin gene expression levels, and is significantly negative correlated with the gene expressions of fibronectin, osteonectin, and α5-integrin (Table 1). It has been reported that the expression E-cadherin is repressed by TWIST1, while the expressions of fibronectin, osteonectin, and α5-integrin are activated by TWIST1 [25]. These results suggest that miR-151-3p might regulate TWIST1 expression and thereby TWIST1-related downstream gene expression.

The data obtained from TCGA revealed that the miR-151 expression levels in breast cancer cells were higher than those in normal tissues (Fig 2A), and no differences were observed in miR-151 levels among tumors of different stages (Fig 2C). Our present in vitro data suggest that miR-151-3p may negatively regulate the migration and invasion of breast cancer cells. The results are consistent with the findings that miR-151 expression was lower in the lymph-node metastases than in their corresponding tumors of breast cancer patients [18]. Recently, upregulation of miR-151 has been reported in esophageal cancer [39] and papillomavirus (HPV)-associated oropharyngeal carcinoma [40]. The up-regulation of miR-151 is found to be associated with poor survival in human HPV-associated oropharyngeal carcinoma [40]. In cholangiocarcinoma, the concomitant dysregulation of both miR-151-3p and miR-126 were reported as factors related to the greatest improvement in overall survival [41]. In addition, an increase in miR-151-3p was identified as one of the most significant differences in miRNA expression for recurrent tumors compared to non-recurrent tumors of the patients with early stage lung adenocarcinomas [42]. Recently, miR-151-3p expression was found to be associated with the ductal carcinoma in situ (DCIS) subtype of breast cancers [43]. These findings suggest that the role of miR-151 in cancer progression seems to be tissue- or cancer type-specific. It is possible that the increased miR-151-3p in breast cancers could be partly involved in the regulation of TWIST1 gene expression at different stages of cancer progression. The TWIST1 expression in breast cancers may be regulated by several mechanisms, including the transcription factors in response to EMT stimulators and miRNAs. These findings need to be further verified using a xenograft model and using the clinical data for immunohistochemical staining of TWIST1 which show anti-correction between TWIST1 and miR-151-3p in breast metastasis.

In conclusion, our results suggested that TWIST1 is the direct target of miR-151-3p and that miR-151-3p may play a negative regulatory role in breast cancer metastasis. Further studies on the regulation of miR-151-3p expression in breast cancer may benefit the development of more specifically targeted therapies for patients with breast cancer metastasis.

## References

[1] Siegel R, Naishadham D, Jemal A. Cancer statistics, 2013. CA Cancer J Clin. 2013; 63: 11–30. 10.3322/caac.21166 23335087

[2] Steeg PS. Targeting metastasis. Nat Rev Cancer. 2016; 16: 201–218. 10.1038/nrc.2016.25 27009393 PMC7055530

[3] Yang J, Mani SA, Donaher JL, Ramaswamy S, Itzykson RA, Come C, et al Twist, a master regulator of morphogenesis, plays an essential role in tumor metastasis. Cell. 2004; 117: 927–939. 10.1016/j.cell.2004.06.006 15210113

[4] Martin TA, Goyal A, Watkins G, Jiang WG. Expression of the transcription factors snail, slug, and twist and their clinical significance in human breast cancer. Ann Surg Oncol. 2005; 12: 488–496. 10.1245/ASO.2005.04.010 15864483

[5] Puisieux A, Valsesia-Wittmann S, Ansieau S. A twist for survival and cancer progression. Br J Cancer. 2006; 94: 13–17. 10.1038/sj.bjc.6602876 16306876 PMC2361066

[6] Xu Y, Hu B, Qin L, Zhao L, Wang Q, Wang Q, et al SRC-1 and Twist1 expression positively correlates with a poor prognosis in human breast cancer. Int J Biol Sci. 2014; 10: 396–403. 10.7150/ijbs.8193 24719557 PMC3979992

[7] Bourgeois P, Stoetzel C, Bolcato-Bellemin AL, Mattei MG, Perrin-Schmitt F. The human H-twist gene is located at 7p21 and encodes a B-HLH protein that is 96% similar to its murine M-twist counterpart. Mamm Genome. 1996; 7: 915–917. 8995765 10.1007/s003359900269

[8] Kalluri R, Weinberg RA. The basics of epithelial-mesenchymal transition. J Clin Invest. 2009; 119: 1420–1428. 10.1172/JCI39104 19487818 PMC2689101

[9] Chou YS, Yang MH. Epithelial-mesenchymal transition-related factors in solid tumor and hematological malignancy. J Chin Med Assoc. 2015; 78: 438–445. 10.1016/j.jcma.2015.05.002 26078096

[10] Vesuna F, van Diest P, Chen JH, Raman V. Twist is a transcriptional repressor of E-cadherin gene expression in breast cancer. Biochem Biophys Res Commun. 2008; 367: 235–241. 10.1016/j.bbrc.2007.11.151 18062917 PMC2696127

[11] Bartel DP. MicroRNAs: genomics, biogenesis, mechanism, and function. Cell. 2004; 116: 281–297. 14744438 10.1016/s0092-8674(04)00045-5

[12] He L, Hannon GJ. MicroRNAs: small RNAs with a big role in gene regulation. Nat Rev Genet. 2004; 5: 522–531. 10.1038/nrg1379 15211354

[13] Garzon R, Calin GA, Croce CM. MicroRNAs in cancer. Annu Rev Med. 2009; 60: 167–179. 10.1146/annurev.med.59.053006.104707 19630570

[14] Iorio MV, Croce CM. microRNA involvement in human cancer. Carcinogenesis 2012; 33: 1126–1133. 10.1093/carcin/bgs140 22491715 PMC3514864

[15] Abba ML, Patil N, Leupold JH, Allgayer H. MicroRNA regulation of epithelial to mesenchymal transition. J Clin Med. 2016; 5: E8 10.3390/jcm5010008 26784241 PMC4730133

[16] Ding J, Huang S, Wu S, Zhao Y, Liang L, Yan M, et al Gain of miR-151 on chromosome 8q24.3 facilitates tumour cell migration and spreading through downregulating RhoGDIA. Nat Cell Biol. 2010; 12: 390–399. 10.1038/ncb2039 20305651

[17] Luedde L. MicroRNA-151 and its hosting gene FAK (focal adhesion kinase) regulate tumor cell migration and spreading of hepatocellular carcinoma. Hepatology 2010; 52: 1164–1166. 10.1002/hep.23854 20812359

[18] Krell J, Frampton AE, Jacob J, Pellegrino L, Roca-Alonso L, Zeloof D, et al The clinico-pathologic role of microRNAs miR-9 and miR-151-5p in breast cancer metastasis. Mol Diagn Ther. 2012; 16: 167–172. 10.2165/11632740-000000000-00000 22489664

[19] Nairismägi ML, Füchtbauer A, Labouriau R, Bramsen JB, Füchtbauer EM. The proto-oncogene TWIST1 is regulated by microRNAs. PLoS One. 2013; 8: e66070 10.1371/journal.pone.0066070 23741524 PMC3669147

[20] Yang MH, Wu MZ, Chiou SH, Chen PM, Chang SY, Liu CJ, et al Direct regulation of TWIST by HIF-1alpha promotes metastasis. Nat Cell Biol. 2008; 10: 295–305. 10.1038/ncb1691 18297062

[21] Wang AM, Huang TT, Hsu KW, Huang KH, Fang WL, Yang MH, et al Yin Yang 1 is a target of microRNA-34 family and contributes to gastric carcinogenesis. Oncotarget. 2014; 5: 5002–5016. 10.18632/oncotarget.2073 24970812 PMC4148117

[22] Huang TT, Ping YH, Wang AM, Ke CC, Fang WL, Huang KH, et al The reciprocal regulation loop of Notch2 pathway and miR-23b in controlling gastric carcinogenesis. Oncotarget. 2015; 6: 18012–18026. 10.18632/oncotarget.4000 26041881 PMC4627232

[23] Hsu KW, Fang WL, Huang KH, Huang TT, Lee HC, Hsieh RH, et al Notch1 pathway-mediated microRNA-151-5p promotes gastric cancer progression. Oncotarget. 2016; 7: 38036–38051. 10.18632/oncotarget.9342 27191259 PMC5122370

[24] Hsu KW, Wang AM, Ping YH, Huang KH, Huang TT, Lee HC, et al Downregulation of tumor suppressor MBP-1 by microRNA-363 in gastric carcinogenesis. Carcinogenesis 2014; 35: 208–217. 10.1093/carcin/bgt285 23975832

[25] Lamouille S, Xu J, Derynck R. Molecular mechanisms of epithelial-mesenchymal transition. Nat Rev Mol Cell Biol. 2014; 15: 178–196. 10.1038/nrm3758 24556840 PMC4240281

[26] Chang YS, Chen WY, Yin JJ, Sheppard-Tillman H, Huang J, Liu YN. EGF receptor promotes prostate cancer bone metastasis by downregulating miR-1 and activating TWIST1. Cancer Res. 2015; 75: 3077–3086. 10.1158/0008-5472.CAN-14-3380 26071255 PMC4909124

[27] Zhou Y, Huang Z, Wu S, Zang X, Liu M, Shi J. miR-33a is up-regulated in chemoresistant osteosarcoma and promotes osteosarcoma cell resistance to cisplatin by down-regulating TWIST. J Exp Clin Cancer Res. 2014; 33: 12 10.1186/1756-9966-33-12 24468065 PMC3974149

[28] Yang L, Yang J, Li J, Shen X, Le Y, Zhou C, et al MircoRNA-33a inhibits epithelial-to-mesenchymal transition and metastasis and could be a prognostic marker in non-small cell lung cancer. Sci Rep. 2015; 5: 13677 10.1038/srep13677 26330060 PMC4556976

[29] Liu S, Cui J, Liao G, Zhang Y, Ye K, Lu T, et al MiR-137 regulates epithelial-mesenchymal transition in gastrointestinal stromal tumor. Tumour Biol. 2014; 35: 9131–9138. 10.1007/s13277-014-2177-5 25027394

[30] Zhu X, Shen H, Yin X, Long L, Xie C, Liu Y, et al miR-186 regulation of Twist1 and ovarian cancer sensitivity to cisplatin. Oncogene. 2016; 35: 323–332. 10.1038/onc.2015.84 25867064

[31] Haga CL, Phinney DG. MicroRNAs in the imprinted DLK1-DIO3 region repress the epithelial-to-mesenchymal transition by targeting the TWIST1 protein signaling network. J Biol Chem. 2012; 287: 42695–42707. 10.1074/jbc.M112.387761 23105110 PMC3522270

[32] Tsukerman P, Yamin R, Seidel E, Khawaled S, Schmiedel D, Bar-Mag T, et al MiR-520d-5p directly targets TWIST1 and downregulates the metastamiR miR-10b. Oncotarget. 2014; 5: 12141–12150. 10.18632/oncotarget.2559 25426550 PMC4323010

[33] Hernandez JM, Elahi A, Clark CW, Wang J, Humphries LA, Centeno B, et al miR-675 mediates downregulation of Twist1 and Rb in AFP-secreting hepatocellular carcinoma. Ann Surg Oncol. 2013; 20 Suppl 3: S625–S635. 23864307 10.1245/s10434-013-3106-3

[34] Li LZ, Zhang CZ, Liu LL, Yi C, Lu SX, Zhou X, et al miR-720 inhibits tumor invasion and migration in breast cancer by targeting TWIST1. Carcinogenesis. 2014; 35: 469–478. 10.1093/carcin/bgt330 24085799

[35] Mitra SK, Hanson DA, Schlaepfer DD. Focal adhesion kinase: in command and control of cell motility. Nat Rev Mol Cell Biol. 2005; 6:56–68. 10.1038/nrm1549 15688067

[36] McLean GW, Carragher NO, Avizienyte E, Evans J, Brunton VG, et al The role of focal-adhesion kinase in cancer—a new therapeutic opportunity. Nat Rev Cancer. 2005; 5: 505–515. 10.1038/nrc1647 16069815

[37] Guidi M, Muiños-Gimeno M, Kagerbauer B, Martí E, Estivill X, Espinosa-Parrilla Y. Overexpression of miR-128 specifically inhibits the truncated isoform of NTRK3 and upregulates BCL2 in SH-SY5Y neuroblastoma cells. BMC Mol Biol. 2010; 11: 95 10.1186/1471-2199-11-95 21143953 PMC3019150

[38] Wei H, Li Z, Wang X, Wang J, Pang W, Yang G, et al microRNA-151-3p regulates slow muscle gene expression by targeting ATP2a2 in skeletal muscle cells. J Cell Physiol. 2015; 230: 1003–1012. 10.1002/jcp.24793 25200835

[39] Gu J, Wang Y, Wu X. MicroRNA in the pathogenesis and prognosis of esophageal cancer. Curr Pharm Des. 2013; 19: 1292–1300. 23092349 10.2174/138161213804805775

[40] Hui AB, Lin A, Xu W, Waldron L, Perez-Ordonez B, Weinreb I, et al Potentially prognostic miRNAs in HPV-associated oropharyngeal carcinoma. Clin Cancer Res. 2013; 19: 2154–2162. 10.1158/1078-0432.CCR-12-3572 23459718

[41] McNally ME, Collins A, Wojcik SE, Liu J, Henry JC, Jiang J, et al Concomitant dysregulation of microRNAs miR-151-3p and miR-126 correlates with improved survival in resected cholangiocarcinoma. HPB (Oxford). 2013; 15: 260–264. 23458262 10.1111/j.1477-2574.2012.00523.x PMC3608979

[42] Edmonds MD, Eischen CM. Differences in miRNA expression in early stage lung adenocarcinomas that did and did not relapse. PLoS One. 2014; 9: e101802 10.1371/journal.pone.0101802 25028925 PMC4100742

[43] Sun EH, Zhou Q, Liu KS, Wei W, Wang CM, Liu XF, et al Screening miRNAs related to different subtypes of breast cancer with miRNAs microarray. Eur Rev Med Pharmacol Sci. 2014; 18: 2783–2788. 25339470

